# Metabolic and pathophysiological characterization of constitutional thinness

**DOI:** 10.1007/s40618-026-02826-2

**Published:** 2026-02-05

**Authors:** Silvio Buscemi, Cristiana Randazzo, Rosa Lo Baido, Sabina La Grutta, Anna Maria Barile, Piero Colombrita, Aurora Ligotino, Serena Cangemi, Silvia Ferro, Roberta Caruso, Martina Lombardo, Carola Buscemi

**Affiliations:** 1 Department of Promozione della Salute, Materno-Infantile, Medicina Interna e Specialistica di Eccellenza (PROMISE), University of Palermo, Palermo, Italy; 2 Clinical Nutrition, Obesity and Metabolic Diseases Unit, University Hospital Policlinico “P. Giaccone”, Palermo, Italy; 3 Department of Experimental Biomedicine, Clinical Neuroscience and Advanced Diagnostic Department (BiND), University of Palermo, Palermo, Italy; 4 Department of Psychology, Educational Science and Human Movement, University of Palermo, Palermo, Italy; 5 Internal Medicine Unit, “V. Cervello” Hospital, Ospedali Riuniti “Villa Sofia-Cervello”, Palermo, Italy; 6 Clinical Nutrition, Obesity and Metabolic Diseases Unit, AOU Policlinico “P. Giaccone”, Piazza delle cliniche 2, Palermo, 90127 Italy

**Keywords:** Constitutional thinness, Anorexia nervosa, Endothelial function, Energy expenditure, Cardiovascular risk

## Abstract

**Purpose:**

Constitutional thinness (CT) is characterized by a persistently low body weight in the absence of eating disorders or overt disease. Distinguishing CT from anorexia nervosa (AN) is often challenging, and the metabolic and cardiovascular features of CT remain incompletely defined. The present study investigated nutritional, metabolic, and cardiovascular parameters in women with CT and compared them with those of women with AN and normal-weight healthy controls.

**Methods:**

Data from 7 women with CT were compared with those from 6 women with AN and 6 normal-weight healthy women serving as controls. The resting metabolic rate (RMR) and endothelial function, assessed by flow-mediated dilatation (FMD), were measured.

**Results:**

Body weight, body mass index, fat mass and fat-free mass (FFM) were comparable between the CT and AN groups and significantly lower than those of the control group. Absolute and FFM-normalized RMR values were significantly higher in the CT group (median [IQR]: 1263 [247] kcal/24 h and 29.2 [3.1] kcal/FFM-kg/24 h) than in the AN group (1046 [272] kcal/24 h and 25.4 [2.7] kcal/FFM-kg/24 h; *P* < 0.001 and *P* < 0.05, respectively), and did not differ from those observed in controls (1317 [221] kcal/24 h and 29.5 [1.2] kcal/FFM-kg/24 h). Flow-mediated dilatation was significantly lower in both the CT (7.2 [2.7] %) and AN (7.6 [6.2] %) groups compared with controls group (14.0 [9.0]%; *P* < 0.05).

**Conclusion:**

These findings indicate that RMR differs between CT and AN, with women with CT exhibiting a metabolic profile distinct from that of AN and similar to that of normal-weight controls. Endothelial dysfunction was observed in both CT and AN, suggesting a potential cardiovascular alteration that warrants further investigation.

## Introduction

Constitutional thinness (CT) is a clinical entity that remains poorly defined from an etiopathogenetic and clinical perspective. Understanding the mechanisms that allow some individuals to maintain a stable low body weight despite apparently increased caloric intake and the absence of concomitant pathologies that cause weight loss could result in the development of better targeted approaches to body weight management as well as a better understanding of the metabolic aspects that regulate body weight. Notably, although individuals with CT often report hypercaloric dietary intake and may have a sedentary lifestyle, their body weight remains remarkably stable over time. This observation has led to the hypothesis that CT may represent a genetically determined, obesity-resistant phenotype. Some studies [1, 2] have shown that a hypercaloric diet is often ineffective for achieving weight gain in individuals with CT. In response to hypercaloric diets, these individuals appear to develop endocrine and metabolic adaptations, including increased energy expenditure and other mechanisms that are not yet fully elucidated [1]. In addition, modulation of appetite- and metabolism-related gut hormones, such as peptide YY (PYY) and glucagon-like peptide 1 (GLP-1), has been reported [3, 4]. However, while these hormonal alterations have been observed in experimental settings, their causal role in the maintenance of low body weight in CT remains speculative, and the underlying mechanisms have yet to be clearly established.

Diagnostic uncertainty represents another major challenge in the clinical management of CT. Both CT and anorexia nervosa (AN) are characterized by low body weight, commonly defined as a body mass index (BMI) < 18.5 kg/m², and misclassification between the two conditions is frequent in clinical practice. Traditionally, CT has been conceptualized as a metabolic or nutritional condition, whereas AN is classified as a psychiatric disorder according to the Diagnostic and Statistical Manual of Mental Disorders (DSM-5) [5, 6]. However, this distinction is increasingly recognized as an oversimplification. Growing evidence suggests partial overlap between the two conditions, with shared biological pathways involving metabolic, neuroendocrine, and gut–brain mechanisms, including potential contributions of the gut microbiome [7].

Conversely, the diagnosis of CT is generally made via exclusion. In particular, the diagnostic features of CT, according to available studies in the literature [1, 8–14] for the differential diagnosis of CT from AN, can be summarized as follows: (a) no psychological or eating disorders or other disorders that could explain the low body weight; (b) a desire to gain body weight; (c) unaltered hormones and markers of nutritional status; and (d) the maintenance of a neutral or positive energy balance.

The cardiovascular implications of CT also remain unclear. While low body weight might intuitively be considered protective, excessive or imbalanced energy intake—regardless of its effect on body weight—could still promote atherogenic processes. Giannini et al. [15] reported increased oxidative stress and endothelial dysfunction in children and adolescents with CT. However, it remains uncertain whether these alterations are primarily driven by growth- and developmental factors or whether they persist into adulthood, potentially conferring long-term cardiovascular risk. To date, no studies have directly compared cardiovascular and metabolic profiles of adult women with CT to those of women with AN.

In the present study, we compared women with CT to women with AN and to normal-weight healthy controls in order to evaluate metabolic and cardiovascular characteristics and to contribute to a more precise clinical and pathophysiological definition of CT.

## Methods

### Participants

Participants who were diagnosed with CT or AN at the Clinical Nutrition, Obesity, and Metabolic Diseases Unit of the University Hospital Policlinico “P. Giaccone” in Palermo (Italy) between November 2018 and June 2020 were included in this study. A dedicated team of doctors and dieticians performed all nutritional evaluations. Some of the data collected were compared with those of a healthy normal-weight control group that consisted of age- and sex-matched individuals. Participants with CT were selected among individuals who reported that they wished to increase their body weight and were unable to do so despite routine normal- or high-calorie food intake. Additional inclusion criteria for participants with CT were female sex, age 18–35 years, BMI < 18.5 kg/m^2^ from pubertal age, stable body weight within the past 2 months, regular dietary habits consisting of at least 3 main meals per day, and a dietary intake of at least 28 kcal/kg of body weight, on the basis of the FFQ validated for the local population. The exclusion criteria for CT were: smoking > 5 cigarettes/day; occurrence of primary or secondary amenorrhea; malignancies; diagnosis of systemic or organ-related diseases, including those that cause malabsorption, hyperthyroidism or hypothyroidism; diagnosis of eating disorders assessed through psychological testing and psychiatric evaluation; and pregnancy. The inclusion criteria for participants with AN were female sex, age 18–35 years, BMI < 18.5 kg/m^2^, and a diagnosis of AN. The exclusion criteria for participants in the AN group were smoking > 5 cigarettes/day; diagnosis of malignancies; diagnosis of systemic or organ-related diseases, including those that cause malabsorption, hyperthyroidism or hypothyroidism; and pregnancy.

A psychiatric specialist interview was performed to determine the presence of an eating disorder, with particular attention to AN, and dedicated tests, such as the Eating Disorders Inventory-3 (EDI-3) [16], the Beck Depression Inventory (BDI-II) [17], the Toronto Structured Interview for Alexithymia (TSIA) [18], and the Somatic Inkblot Series (SIS) [19], were administered.

The institutional ethics committee (“Palermo 1” of the Policlinico “P. Giaccone” University Hospital, 10 September 2018, ref: 08/2018) approved the study protocol, and each participant signed an approved informed consent form.

### Anthropometric and clinical measurements

Body composition was estimated as fat mass (FM, percentage of body weight) and fat-free mass (FFM, kg) using bioelectrical impedance analysis (BIA), as previously described [20] [BIA-101 Anniversary, Akern Srl, Florence, Italy], to obtain body resistance (R; ohm), reactance (Xc, ohm), and phase angle [PA degrees 5 arctan (Xc/R) 3 (180/p)]. The use of crude BIA measures, such as the phase angle (PA), has received increased attention, as these may be plausible indicators of intra- and extracellular hydration and nutritional status [21]. The body circumference was measured at the umbilicus (waist circumference) and at the most prominent buttock level (hip circumference). The waist-to-hip ratio (WHR) was used as an indirect index of body fat distribution. The volumes of the abdominal visceral and subcutaneous adipose tissues was also measured by means of high-resolution B-mode ultrasound (Envisor G5; Philips, US) [22]. Transverse scans were obtained 5 cm above the umbilicus along the xipho-umbilical line. A 10-MHz linear probe was used to measure the distance between the cutis and the conjunction of rectus muscles at the linea alba (cutis-rectis thickness; CR) as a measure of subcutaneous fat. A 3.5-MHz convex probe was used to measure the distance between the linea alba and the anterior wall of the abdominal aorta (rectis-aorta thickness, or RA) as a measure of visceral abdominal fat. The RA-to-CR ratio (RA/CR) is also considered an indirect measure of body fat distribution. Muscle strength was measured with a hand grip test using a hydraulic hand dynamometer (model no. SH5001; Jamar, Saehan, Republic of Korea). The participants were instructed to perform a maximal isometric contraction. The test was repeated within 15 to 20 s for each hand, and the average value (kg) of the three tests was used for the analysis [23]. Habitual dietary intake was assessed using a validated food frequency questionnaire (FFQ) specifically designed for the local population [24]. The participants were required to report their typical dietary habits over the previous year, including their meal patterns and food choices. Data from the FFQ were analyzed to determine overall dietary characteristics. Habitual physical activity level was investigated using a specific questionnaire validated for the local population that describes 4 levels (very low = 1, low = 2, moderate = 3, high = 4) of physical activity [25].

### Indirect calorimetry

The resting metabolic rate (RMR) was determined using the indirect calorimetry method, as previously described [26], and a ventilated hood system (Quark RMR; Cosmed, Rome, Italy). The device was equipped with an infrared analyzer for carbon dioxide measurement (VCO2) and a zirconium cell analyzer for oxygen measurement (VO2). The analyzers were calibrated before each test using gases with a known percentage of oxygen and carbon dioxide. The data were obtained from approximately 30–60 min of stable measurements, and the average intrasubject variability in the RMR was 3.9%. The RMR was calculated using the Weir equation [27] and was expressed both in absolute terms (kcal/24 h) and normalized to the FFM value (kcal/kg-FFM • 24 h). The respiratory quotient (RQ; VCO2/VO2), an indirect measure of both carbohydrate and lipid oxidation, was obtained. With respect to the substrate oxidation measurement [28], the urea nitrogen excreted in the urine over the last 12 h is assumed to be derived from protein oxidation; the grams of oxidized proteins were obtained by multiplying the amount (g) of ureic nitrogen by 6.25. Since the quantity of O_2_ necessary to oxidize one gram of protein and the amount of CO_2_ produced are known, the nonprotein RQ (NPRQ) was obtained by subtracting these estimated volumes from the measured volumes; thus, this value reflected only the relative proportions of lipid and carbohydrate oxidation.

### Endothelial function

The flow-mediated dilatation (FMD) of the brachial artery was assessed as a measure of endothelial function using a high-resolution ultrasound linear probe (10 MHz, Sonoline G50 Philips, US), as previously described [29]. Briefly, a sphygmomanometer, cuffed at 220–250 mmHg 2 cm below the antecubital fossa, induces reactive hyperemia by occluding the artery for 300 s. Real-time computed video analysis of B-mode ultrasound images (FMD Studio; Institute of Physiology CNR; Pisa, Italy) recorded variations in brachial artery diameter. The baseline vessel size was determined as the mean of measurements obtained during the first minute. FMD was calculated as the maximum percentage increase in brachial artery diameter over the baseline. Endothelium-independent dilatation was assessed after the administration of 300 µg sublingual glyceryl–trinitrate (GTN). All flow-mediated dilation and glyceryl trinitrate dilatation assessments were performed by the same operator; ultrasound images were video recorded and analyzed by a trained reader. The intra-observer coefficient of variation for flow-mediated dilation was 2.9% in our laboratory.

### Laboratory exams

Fasting blood samples were collected from all participants after an overnight fast of at least 8 h. Fasting plasma glucose (FPG), total cholesterol, high-density lipoprotein cholesterol (HDL-c), triglyceride (Tg), uric acid, and creatinine concentrations were measured using standard clinical chemistry methods (Glucosio HK UV; Colesterolo tot. Mod P/D; Colesterolo HDL gen 3 mod P/917; Trigliceridi; Acido urico MOD P/917; Creatinina enzimatica; Roche Diagnostics; Monza, Italy). Serum low-density lipoprotein cholesterol (LDL-c) concentrations were calculated using Friedewald’s formula, and HOMA-I values were calculated as described by Matthews et al. [30].

#### Statistical analysis

Statistical analysis was performed using Systat (Windows version 13.0; San Jose, CA, USA). Given the small sample size in each group (*n* = 6–7), continuous variables are reported as median and interquartile range (IQR). Differences among the three groups were assessed using the Kruskal–Wallis test. When the overall test was significant, post hoc pairwise comparisons were conducted using the Conover–Inman procedure with Holm correction for multiple testing. A two-sided p value < 0.05 was considered statistically significant.

## Results

In this study, one woman with AN and one woman in the control group refused to participate in the study. Finally, we recruited 7 women with CT, 6 women with AN and 6 controls. The physical and clinical characteristics of the participants in the three groups are presented in Table 1. Notably, the physical characteristics of the AN and CT groups did not significantly differ. Compared with the control group, both the AN group and the CT group exhibited lower body weights and FMs with less subcutaneous fat. Data related to dietary habits, energy expenditure and basal substrate oxidation are presented in Table 2. The habitual intake of energy and macronutrients did not differ among the three groups.

**Table 1:** Physical and clinical characteristics of the women with anorexia nervosa (AN) and constitutional thinness (CT)

	Groups			K-W test *P*
	**Control** (*n* = 6)	**AN** (*n* = 6)	**CT** (*n* = 7)	
Age (years)	30 (2)	23 (6)	28 (5)	0.36
Body weight (kg)	57.0 (7.7)^∗, **^	47.5 (4.1)	46.4 (5.1)	< 0.05
BMI (kg/m^2^)	20.8 (2.0)^#, ••^	17.1 (0.3)	17.9 (0.5)	< 0.005
Body circumferences:				
Waist (cm)	80.0 (6.7)^•, ••^	65.0 (1.5)	66.0 (1.9)	< 0.05
Hip (cm)	95.0 (5.2)^•, ∗∗^	85.0 (5.0)	88.5 (7.4)	< 0.05
Waist to hip ratio	0.81 (0.08)^*, **^	0.76 (0.03)	0.75 (0.07)	< 0.05
Bioimpedance analysis:				
Resistance (Ohm)	649 (90)	733 (75)	673 (36)	0.12
Reactance (Ohm)	69 (13)	82 (13)	75 (8)	0.28
Phase angle (°)	7.3 (1.2)	6.6 (0.8)	6.4 (0.4)	0.50
Fat mass (%)	21.1 (4.8)^*,**^	15.0 (1.5)	14.8 (2.7)	< 0.05
Fat free mass (kg)	46.4 (7.5)	40.7 (3.2)	40.1 (2.2)	0.31
Ultrasound thicknesses:				
Cutis-rectis (mm)	18 (14)	4 (1)	6 (2)	0.47
Rectis-aorta (mm)	3 (1)	4 (1)	2 (1)	0.06
Hand-grip test (kg)	30 (3)	25 (5)	24 (5)	0.22
Blood concentrations of:				
Glucose (mg/dl)	83 (8)	81 (9)	80 (6)	0.68
Cholesterol (mg/dl)	181 (31)	164 (31)	162 (26)	0.60
Triglycerides (mg/dl)	89 (13)	85 (29)	60 (57)	0.63
HDL-c (mg/dl)	67 (16)	54 (9)	66 (32)	0.43
LDL-c (mg/dl)	96 (18)	99 (23)	80 (19)	0.30
Uric acid (mg/dl)	4.1 (0.3)	3.3 (0.5)	3.4 (0.9)	0.14
Insulin (mU/ml)	6.6 (2.7)	5.0 (3)	6.1 (1.7)	0.81
HOMA-I	1.3 (0.8)	0.9 (0.7)	1.2 (0.5)	0.72
C-reactive protein (mg/l)	2.2 (0.9)	0.6 (0.7)	0.4 (0.3)	0.24

Median; interquartile range in parenthesis; KW, Kruskal-Wallis test; Pairwise comparisons: ^*^P < 0.05 vs. AN group, ^**^P < 0.05 vs. CT group, ^•^*P* < 0.01 vs. AN group, ^••^*P* < 0.01 vs. CT group, ^#^*P* < 0.001 vs. AN group

BMI, body mass index; HDL-c, high-density lipoprotein cholesterol; HOMA-I, homeostatic model assessment index; LDL-c, low-density lipoprotein cholesterol

**Table 2:** Dietary habits, energy expenditure and basal substrate oxidation in women with anorexia nervosa (AN) and constitutional thinness (CT)

	Groups			K-W test
	**Control** (*n* = 6)	**AN** (*n* = 6)	**CT** (*n* = 7)	*P*
Habitual intake of:				
Energy (kcal/24 h)	1425 (555)	1225 (712)	1571 (226)	0.91
Carbohydrates (% energy intake)	48.1 (14.1)	47.6 (19)	55.0 (14.9)	0.73
Lipids (% energy intake)	32.2 (7.1)	30.7 (16.1)	25.2 (13.3)	0.91
Proteins (% energy intake)	19.7 (6.9)	21.7 (6.3)	19.8 (2.5)	0.56
Proteins (g/24 h)	70 (7)	72.8 (35)	74.9 (19)	0.99
Physical activity (score)	3.0 (0.8)	2.0 (1.5)	2.0 (1.0)	0.15
Resting metabolic rate:				
kcal/24 h	1317 (221) ^#^	1046 (272)	1263 (247)^#^	< 0.05
kcal/(kg FFM•24 h)	29.5 (1.2) ^•^	25.4 (2.7)	29.2 (3.1) ^•^	< 0.05
Basal substrate oxidation:				
NPRQ	0.74 (0.05)	0.80 (0.04)	0.73 (0.08)	0.08
Carbohydrates (%)	16.6 (12.3)	26.5 (32.2)	8.5 (16.1)	0.20
Lipids (%)	55.5 (5.0)	56.9 (24.1)	64.6 (10.1)	0.26
Proteins (%)	27.9 (7.2)	22.0 (11.5)	23.3 (8.7)	0.36

Median, interquartile range in parenthesis; KW, Kruskal-Wallis test; Pairwise comparisons: ^•^*P* < 0.05 vs. AN group, ^#^*P* < 0.001 vs. AN group

FFM, fat-free mass; NPRQ, nonprotein respiratory quotient

**Fig. 1:**
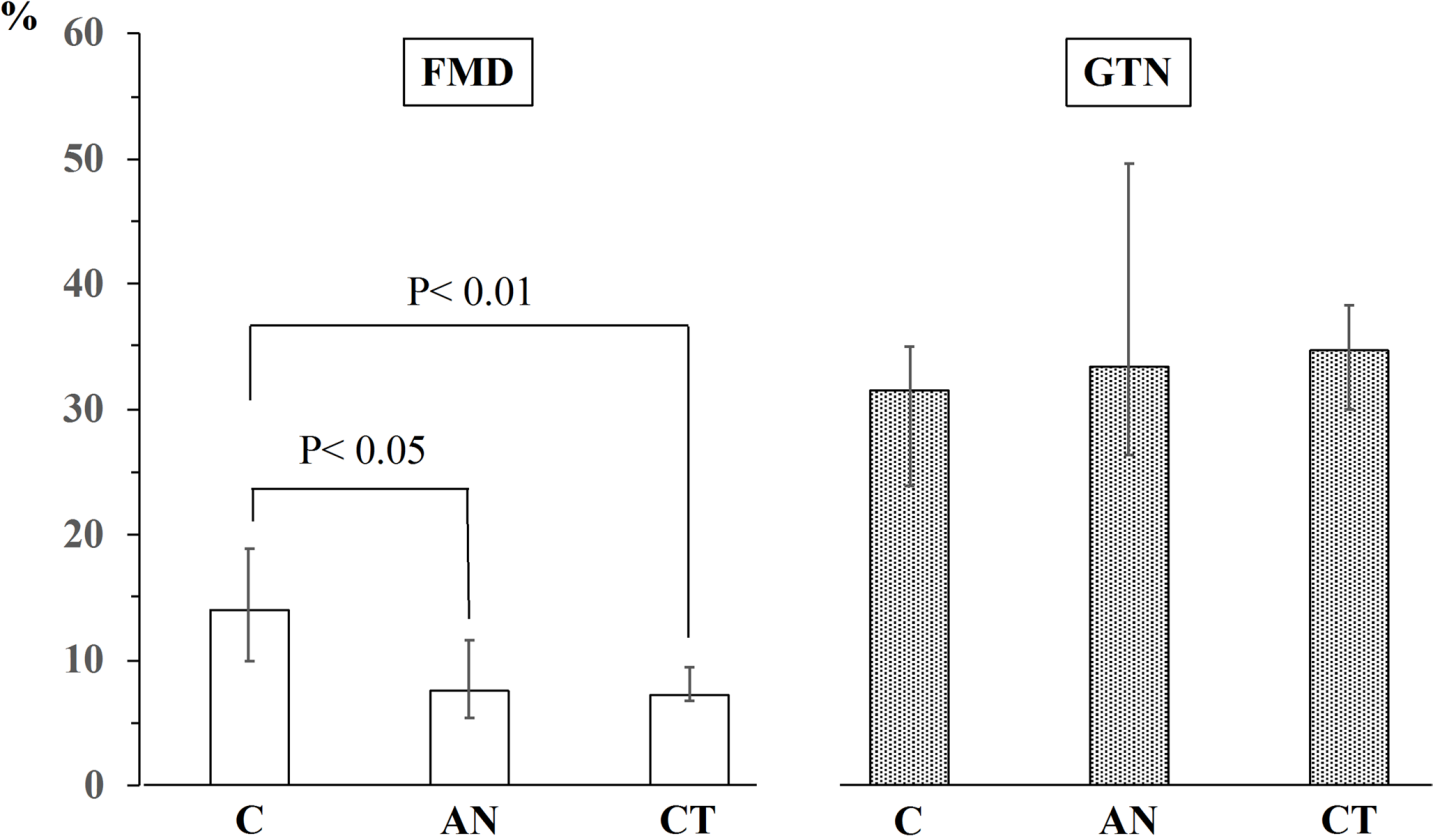
Endothelial function assessed by flow-mediated and glyceryl-trinitrate–mediated dilation across control, anorexia nervosa, and constitutional thinness groups. Endothelial function was assessed as flow-mediated dilation (FMD) and endothelium-independent dilation following the administration of 300 μg sublingual glyceryl trinitrate (GTN) of the brachial artery in the control (C), anorexia nervosa (AN), and constitutional thinness (CT) groups. Bars represent median values and error bars indicate the interquartile range (25th–75th percentiles). Group comparisons were performed using the Kruskal–Wallis test followed by Conover–Inman post hoc analysis with Holm correction

Both the absolute and FFM-normalized RMR values of the AN group were significantly lower than those of the CT group and the control group. The FMD and GTN data are presented in Fig. 1. Compared with those in the control group (median value 14.0%; IQR 9.0%) participants in the AN (median value 7.6%; IQR 6.2%) and CT (median value 7.2%; IQR 2.7%) groups had lower FMD values (Kruskal-Wallis test: *P* < 0.05). The GTN was not significantly different among the three groups (Kruskal-Wallis test: *P* = 0.60).

## Discussion

The results of this study suggest that women with CT are psychologically and clinically healthy. Therefore, the assessment of anthropometric and psychological parameters might be sufficient to allow it to be differentially diagnosed with eating disorders that also cause thinness. We did not observe clear evidence of high energy expenditure that would explain the thinness seen in the context of high habitual caloric intake. However, some data support this possibility. First, both the absolute and FFM-normalized RMRs were significantly greater in the CT group than in the AN group, but the values in the CT group were comparable to those of the control group. These findings are consistent with the possibility that in women with AN, caloric restriction, and consequently a negative energy balance, promote a reduction in energy expenditure that favors energy conservation, maintains energy balance, and slows weight loss [31]. Therefore, in women with CT, the value of the RMR was normal since these women did not experience a reduction in habitual caloric intake. These findings indicate that resting metabolic rate normalized for fat-free mass differs between constitutional thinness and anorexia nervosa. Although not intended as a diagnostic tool and despite partial overlap of individual values, this parameter may represent a physiological feature that contributes to distinguishing these two conditions when interpreted in conjunction with clinical assessment. Additionally, compared with the control group, the CT group maintained a significantly lower body weight despite a similar resting metabolic rate, suggesting a reduced energetic efficiency in these individuals. Although data concerning energy expenditure in constitutional thinness are scarce, this observation is consistent with the hypothesis of a condition characterized by increased energy dissipation [32–34]. However, resting metabolic rate represents only one, albeit the major, component of total daily energy expenditure. Therefore, total energy expenditure may not be fully captured by resting metabolic rate alone. In this regard, although structured physical activity levels did not differ between groups in the present study, non-exercise activity thermogenesis (NEAT), which includes spontaneous and unintentional movements such as fidgeting or postural adjustments, was not specifically assessed and may have been underestimated. Increased NEAT could therefore contribute to higher total energy expenditure in individuals with constitutional thinness and help explain the discrepancy between stable low body weight and apparently normal energy intake. Other components of daily energy expenditure not investigated in this study, including postprandial thermogenesis, may also play a role. These observations align with a recent review [35] indicating that constitutional thinness represents a distinct phenotype with unique energy regulation profiles and metabolic features that challenge traditional concepts of energy balance. Nevertheless, these considerations remain speculative and require confirmation in future studies.

The assessment of habitual dietary intake in individuals with anorexia nervosa represents a major methodological challenge, as no currently available approach can provide fully accurate or unbiased estimates in this population. Self-reported tools, including food frequency questionnaires, are particularly prone to recall and reporting bias in individuals with eating disorders. However, in the absence of more reliable alternatives for the evaluation of habitual intake, a food frequency questionnaire validated for our geographical area was used in the present study. The similar reported energy intake observed across groups is therefore unlikely to reflect true equivalence in dietary intake and should be interpreted with caution. Nevertheless, it is noteworthy that the constitutional thinness group reported a habitual energy intake comparable to that of the control group. Although this finding should not be overinterpreted given the limitations of dietary self-report, it is consistent with the study hypothesis and may suggest the presence of metabolic or physiological mechanisms allowing constitutional thinness women to maintain a significantly lower body weight despite an apparently similar energy intake. It should also be noted that, although not an inclusion criterion, all patients with anorexia nervosa who participated in the study were of the restricting subtype, which may partially limit heterogeneity but does not eliminate the intrinsic limitations of dietary self-report in this condition.

The present study cannot provide direct insights into the pathophysiological mechanisms underlying constitutional thinness. Among the proposed hypotheses, an increased contribution of brown or beige adipose tissue has been suggested. However, in the absence of direct imaging or functional assessment in the present study, the role of brown adipose tissue should be considered speculative and mainly supported by previous literature. A small PET study reported an association between brown adipose tissue expansion and higher resting metabolic rate in women with constitutional thinness [32], and brown adipose tissue has been shown to be inversely associated with body mass index and potentially protective against obesity [36, 37]. Nonetheless, further studies are required to clarify the relative contribution of these mechanisms.

Furthermore, an interesting finding, despite not statistically significant, is that women with CT oxidize more fatty acids under basal conditions; these data are in agreement with the possibility that the CT group may have had a negative energy balance in the presence of adequate energy intake. On the contrary, even if not statistically significant, we found that compared with women in the control group, women in the CT group tended to habitually consume a lower quantity of fats and a higher quantity of carbohydrates. Therefore, a negative balance between fat intake and fat oxidation occurs, which is a characteristic that is in line with the condition of leanness [38]. Similarly, Ling et al. demonstrated that adipocytes from individuals with CT are smaller in size than adipocytes from normal-weight individuals and that the mitochondria in those with CT exhibit more intense activity and more marked lipid oxidation activity compared with mitochondria in controls, which was also confirmed through indirect calorimetry [39].

Epidemiological evidence indicates an increased mortality risk even in BMI classes indicative of thinness. In line with this observation, we found that, compared with the control group, both CT and AN were characterized by endothelial dysfunction, as demonstrated by significantly lower FMD values, despite preserved endothelium-independent vasodilation, as assessed by GTN. Endothelial dysfunction represents a key early event in the development of atherosclerosis [40], and FMD is considered the reference non-invasive method for the in vivo assessment of endothelial function in humans [41]. Several studies have shown that reduced FMD is associated with increased cardiovascular events and reduced survival [42–44]. Therefore, the presence of impaired endothelial function in women with constitutional thinness suggests the possibility of an increased cardiovascular risk and highlights the need for careful clinical evaluation and long-term follow-up in this population. However, these findings should not be interpreted as sufficient to define constitutional thinness as a disease requiring treatment, but rather as evidence of a physiological alteration that may have relevant clinical implications and deserves further investigation.

Different mechanisms may underlie endothelial dysfunction in constitutional thinness and anorexia nervosa. In constitutional thinness, excessive substrate oxidation may promote oxidative stress, potentially contributing to endothelial and metabolic stress, whereas in anorexia nervosa, antioxidant deficiencies may play a predominant role.

This study has several limitations, but the main limitation is likely the small sample size. However, the incidence of well-defined cases of CT is relatively low, and therefore, it is difficult to include a substantial number of case studies. Furthermore, the inclusion of individuals with anorexia nervosa in studies similar to this one is difficult. However, the merits of this study are that it considered multiple nutritional, metabolic, and cardiovascular aspects in the same subjects and compared cases of CT with difficult-to-interpret conditions such as anorexia nervosa. Only well-coordinated multicenter studies will be able to obtain data from larger case series. Another limitation of this study is the use of BIA to assess body composition. Bioelectrical impedance analysis is widely used for the assessment of body composition; however, its validity in very lean populations deserves consideration. Previous studies have shown that BIA tends to underestimate fat-free mass in individuals with low body mass index, while overestimating it in subjects with higher BMI, likely due to alterations in hydration status and body geometry [45]. Importantly, this potential source of systematic error is unlikely to explain the present findings regarding resting metabolic rate normalized for fat-free mass (RMR/FFM). Indeed, the anorexia nervosa and constitutional thinness groups were characterized by comparable BMI values; therefore, any BIA-related bias would be expected to affect both groups in a similar manner. Despite this, RMR normalized for fat-free mass was significantly different between the two groups, suggesting that the observed difference is unlikely to be attributable solely to measurement-related bias and may reflect a genuine difference in resting metabolic rate between the two conditions. Nevertheless, confirmation of these findings using reference techniques such as dual-energy X-ray absorptiometry would be desirable in future studies.

In conclusion, the results of this study support the hypothesis that individuals with CT exhibit a distinct energy regulation profile, particularly when compared with individuals with AN. Endothelial dysfunction seems to characterize individuals with CT or AN. Further studies are necessary to confirm these observations and to better understand the mechanisms responsible for the energy dissipating condition in CT.

## Data Availability

The datasets generated during this study are available from the corresponding author upon reasonable request for 2 years following the date of publication.

## Abbreviations

AN: Anorexia nervosa
BIA: Bioelectrical impedance analysis
BMI: Body mass index
CR: Cutis-rectis thickness
CT: Constitutional thinness
FFM: Fat-free mass
FM: Fat mass
FFQ: Food frequency questionnaire
FMD: Flow-mediated dilatation
FPG: Fasting plasma glucose
GTN: Glyceryl–trinitrate
HDL: High-density lipoprotein
LDL: Low-density lipoprotein
NPRQ: Nonprotein respiratory quotient
PA: Phase angle
RA: Rectis-aorta thickness
RMR: Resting metabolic rate
TG: Triglyceride
WHR: Waist-to-hip ratio

## References

[1] Germain N, Galusca B, Caron-Dorval D, Martin JF, Pujos-Guillot E, Boirie Y et al (2014) Specific appetite, energetic and metabolomics responses to fat overfeeding in resistant-to-body weight-gain constitutional thinness. Nutr Diabetes 4(7):e126. 10.1038/nutd.2014.17 25027794 10.1038/nutd.2014.17 PMC5189928

[2] Ling Y, Galusca B, Hager J, Feasson L, Valsesia A, Epelbaum J et al (2016) Rational and design of an overfeeding protocol in constitutional thinness: understanding the physiology, metabolism and genetic background of resistance to weight gain. Ann Endocrinol 77:563–9. 10.1016/j.ando.2016.06.001 10.1016/j.ando.2016.06.001 27424229

[3] Batterham RL, Cowley MA, Small CJ, Herzog H, Cohen MA, Dakin CL et al (2002) Gut hormone PYY(3–36) physiologically inhibits food intake. Nature 418(6898):650–4. 10.1038/nature00887 12167864 10.1038/nature00887

[4] Holst JJ (2007) The physiology of glucagon-like peptide 1. Physiol Rev 87:1409–1439. 10.1152/physrev.00034.2006 17928588 10.1152/physrev.00034.2006

[5] Estour B, Galusca B, Germain N (2014) Constitutional thinness and anorexia nervosa: a possible misdiagnosis? Front Endocrinol (Lausanne) 5:175. 10.3389/fendo.2014.00175 25368605 10.3389/fendo.2014.00175 PMC4202249

[6] American Psychiatric Publishing, American Psychiatric Association (2013) Diagnostic and Statistical Manual of Mental Disorders, DSM-5, 5th edition. Arlington, Virgina

[7] Fan Y, Støving RK, Berreira Ibraim S, Hyötyläinen T, Thirion F, Arora T et al (2023) The gut microbiota contributes to the pathogenesis of anorexia nervosa in humans and mice. Nat Microbiol 8:787–802. 10.1038/s41564-023-01355-5 37069399 10.1038/s41564-023-01355-5 PMC10159860

[8] Estour B, Marouani N, Sigaud T, Lang F, Fakra E, Ling Y et al (2017) Differentiating constitutional thinness from anorexia nervosa in DSM 5 era. Psychoneuroendocrinology 84:94–100. 10.1016/j.psyneuen.2017.06.015 28692876 10.1016/j.psyneuen.2017.06.015

[9] Germain N, Viltart O, Loyens A, Bruchet C, Nadin K, Wolowczuk I et al (2016) Interleukin-7 plasma levels in human differentiate anorexia nervosa, constitutional thinness and healthy obesity. PLoS One 11(9):e0161890. 10.1371/journal.pone.0161890 27611669 10.1371/journal.pone.0161890 PMC5017702

[10] Tolle V, Kadem M, Bluet-Pajot MT, Frere D, Foulon C, Bossu C, Dardennes R et al (2003) Balance in ghrelin and leptin plasma levels in anorexia nervosa patients and constitutionally thin women. J Clin Endocrinol Metab 88(1):109–16. 10.1210/jc.2002-020645 12519838 10.1210/jc.2002-020645

[11] Germain N, Galusca B, Grouselle D, Frere D, Tolle V, Zizzari P (2009) Ghrelin/obestatin ratio in two populations with low bodyweight: constitutional thinness and anorexia nervosa. Psychoneuroendocrinology 34:413–419. 10.1016/j.psyneuen.2008.10.001 18995969 10.1016/j.psyneuen.2008.10.001

[12] Marra M, Caldara A, Montagnese C, De Filippo E, Pasanisi F, Contaldo F et al (2009) Bioelectrical impedance phase angle in constitutionally lean females, ballet dancers and patients with anorexia nervosa. Eur J Clin Nutr 63:905–8. 10.1038/ejcn.2008.54 19002201 10.1038/ejcn.2008.54

[13] Germain N, Galusca B, Le Roux CW, Bossu C, Ghatei MA, Lang F et al (2007) Constitutional thinness and lean anorexia nervosa display opposite concentrations of peptide YY, glucagon-like peptide 1, ghrelin, and leptin. Am J Clin Nutr 85:967–71. 10.1093/ajcn/85.4.967 17413094 10.1093/ajcn/85.4.967

[14] Bossu C, Galusca B, Normand S, Germain N, Collet P, Frere D et al (2007) Energy expenditure adjusted for body composition differentiates constitutional thinness from both normal subjects and anorexia nervosa. Am J Physiol Endocrinol Metab 292:E132-7. 10.1152/ajpendo.00241.2006 16912058 10.1152/ajpendo.00241.2006

[15] Giannini C, de Giorgis T, Scarinci A, Cataldo I, Marcovecchio ML, Chiarelli F et al (2009) Increased carotid intima-media thickness in pre-pubertal children with constitutional leanness and severe obesity: the speculative role of insulin sensitivity, oxidant status, and chronic inflammation. Eur J Endocrinol 161:73–80. 10.1530/EJE-09-0042 19423560 10.1530/EJE-09-0042

[16] Clausen L, Rosenvinge JH, Friborg O, Rokkedal K (2011) Validating the eating disorder inventory-3 (EDI-3): a comparison between 561 female eating disorders patients and 878 females from the general population. J Psychopathol Behav Assess 33:101–110. 10.1007/s10862-010-9207-4 21472023 10.1007/s10862-010-9207-4 PMC3044826

[17] Beck AT (1961) An inventory for measuring depression. Arch Gen Psychiatry 4:561. 10.1001/archpsyc.1961.01710120031004 13688369 10.1001/archpsyc.1961.01710120031004

[18] Caretti V, Porcelli P, Solano L, Schimmenti A, Bagby RM, Taylor GJ (2011) Reliability and validity of the Toronto Structured Interview for Alexithymia in a mixed clinical and nonclinical sample from Italy. Psychiatry Res 187:432–6. 10.1016/j.psychres.2011.02.015 21396720 10.1016/j.psychres.2011.02.015

[19] Cassell WA (1980) Body symbolism and the somatic inkblot series. Paperback. Northern Lights Pub Ed

[20] Buscemi S, Blunda G, Maneri R, Verga S (1998) Bioelectrical characteristics of type 1 and type 2 diabetic subjects with reference to body water compartments. Acta Diabetol 35:220–223. 10.1007/s005920050135 9934822 10.1007/s005920050135

[21] Norman K, Stobäus N, Pirlich M, Bosy-Westphal A (2012) Bioelectrical phase angle and impedance vector analysis-clinical relevance and applicability of impedance parameters. Clin Nutr 31:854–61. 10.1016/j.clnu.2012.05.008 22698802 10.1016/j.clnu.2012.05.008

[22] Armellini F, Zamboni M, Robbi R, Todesco T, Rigo L, Bergamo-Andreis IA et al (1993) Total and intra-abdominal fat measurements by ultrasound and computerized tomography. Int J Obes Relat Metab Disord 17(4):209–214 8387970

[23] Reddon JR, Stefanyk WO, Gill DM, Renney C (1985) Hand dynamometer: effects of trials and sessions. Percept Mot Skills 61(3 Pt 2):1195–1198. 10.2466/pms.1985.61.3f.1195 4094860 10.2466/pms.1985.61.3f.1195

[24] Buscemi S, Rosafio G, Vasto S, Massenti FM, Grosso G, Galvano F et al (2015) Validation of a food frequency questionnaire for use in Italian adults living in Sicily. Int J Food Sci Nutr 66:426–38. 10.3109/09637486.2015.1025718 25830946 10.3109/09637486.2015.1025718

[25] Buscemi S, Corleo D, Vasto S, Buscemi C, Massenti MF, Nuzzo D et al (2018) Factors associated with circulating concentrations of irisin in the general population cohort of the ABCD study. Int J Obes 42:398–404. 10.1038/ijo.2017.255 10.1038/ijo.2017.255 29027533

[26] Buscemi S, Verga S, Caimi G, Cerasola G (2005) Low relative resting metabolic rate and body weight gain in adult Caucasian Italians. Int J Obes 29:287–291. 10.1038/sj.ijo.0802888 10.1038/sj.ijo.0802888 15672106

[27] Weir JB (1949) New methods for calculating metabolic rate with special reference to protein metabolism. J Physiol 109(1–2):1–9. 10.1113/jphysiol.1949.sp004363 15394301 10.1113/jphysiol.1949.sp004363 PMC1392602

[28] Ferrannini E (1988) The theoretical bases of indirect calorimetry: a review. Metabolism 37:287–301. 10.1016/0026-0495(88)90110-2 3278194 10.1016/0026-0495(88)90110-2

[29] Buscemi S, Verga S, Tranchina MR, Cottone S, Cerasola G (2009) Effects of hypocaloric very-low-carbohydrate diet vs. Mediterranean diet on endothelial function in obese women. Eur J Clin Invest 39:339–47. 10.1111/j.1365-2362.2009.02091.x 19302563 10.1111/j.1365-2362.2009.02091.x

[30] Matthews DR, Hosker JP, Rudenski AS, Naylor BA, Treacher DF, Turner RC (1985) Homeostasis model assessment: insulin resistance and beta-cell function from fasting plasma glucose and insulin concentrations in man. Diabetologia 28:412–9. 10.1007/BF00280883 3899825 10.1007/BF00280883

[31] Rothwell NJ, Stock MJ (1979) A role for brown adipose tissue in diet-induced thermogenesis. Nature 281:31–35. 10.1038/281031a0 551265 10.1038/281031a0

[32] Pasanisi F, Pace L, Fonti R, Marra M, Sgambati D, De Caprio C et al (2013) Evidence of brown fat activity in constitutional leanness. J Clin Endocrinol Metab 98:1214–8. 10.1210/jc.2012-2981 23393181 10.1210/jc.2012-2981

[33] Marugán de Miguelsanz JM, Redondo del Río MP, Alonso-Franch M, Calvo Romero C, Torres Hinojal MC (2011) Increased resting energy expenditure by fat-free mass in children and teenagers with constitutional leanness. Nutr Hosp 26:589–593. 10.1590/S0212-16112011000300023 21892579 10.1590/S0212-16112011000300023

[34] Marra M, Pasanisi F, Montagnese C, De Filippo E, De Caprio C, de Magistris L et al (2007) Bmr variability in women of different weight. Clin Nutr 26:567–72. 10.1016/j.clnu.2007.03.006 17517450 10.1016/j.clnu.2007.03.006

[35] Bailly M, Thivel D, Isacco L, Verney J (2025) Unique energy profile associated with the persistent thin phenotype. Annu Rev Nutr 45:65–91. 10.1146/annurev-nutr-111824-014837 40327537 10.1146/annurev-nutr-111824-014837

[36] Cypess AM, Lehman S, Williams G, Tal I, Rodman D, Goldfine AB et al (2009) Identification and importance of brown adipose tissue in adult humans. N Engl J Med 360:1509–17. 10.1056/NEJMoa0810780 19357406 10.1056/NEJMoa0810780 PMC2859951

[37] Franssens BT, Hoogduin H, Leiner T, van der Graaf Y, Visseren FLJ (2017) Relation between brown adipose tissue and measures of obesity and metabolic dysfunction in patients with cardiovascular disease. J Magn Reson Imaging 46:497–504. 10.1002/jmri.25594 28130811 10.1002/jmri.25594

[38] Buscemi S, Verga S, Caimi G, Bompiani GD (1994) Postabsorptive lipid oxidation and obesity: effect of massive weight reduction. In: Ditschuneit H, Gries FA, Hauner H, Schusdziarra V, Wechsler JG (eds) Obesity in Europe 1993. John Libbey, London, pp 453–457

[39] Ling Y, Carayol J, Galusca B, Canto C, Montaurier C, Matone A et al (2019) Persistent low body weight in humans is associated with higher mitochondrial activity in white adipose tissue. Am J Clin Nutr 110:605–16. 10.1093/ajcn/nqz144 31374571 10.1093/ajcn/nqz144 PMC6736451

[40] Buscemi S, Rosafio G, Arcoleo G, Mattina A, Canino B, Montana M et al (2012) Effects of red orange juice intake on endothelial function and inflammatory markers in adult subjects with increased cardiovascular risk. Am J Clin Nutr 95:1089–95. 10.3945/ajcn.111.031088 22492368 10.3945/ajcn.111.031088

[41] Thijssen DH, Black MA, Pyke KE, Padilla J, Atkinson G, Harris RA et al (2011) Assessment of flow-mediated dilation in humans: a methodological and physiological guideline. Am J Physiol Heart Circ Physiol 300:H2–12. 10.1152/ajpheart.00471.2010 20952670 10.1152/ajpheart.00471.2010 PMC3023245

[42] Rossi R, Nuzzo A, Origliani G, Modena MG (2008) Prognostic role of flow-mediated dilation and cardiac risk factors in post-menopausal women. J Am Coll Cardiol 51:997–1002. 10.1016/j.jacc.2007.11.044 18325438 10.1016/j.jacc.2007.11.044

[43] Yeboah J, Crouse JR, Hsu FC, Burke GL, Herrington DM (2007) Brachial flow-mediated dilation predicts incident cardiovascular events in older adults: the cardiovascular health study. Circulation 115:2390–2397. 10.1161/CIRCULATIONAHA.106.678276 17452608 10.1161/CIRCULATIONAHA.106.678276

[44] Shechter M, Shechter A, Koren-Morag N, Feinberg MS, Hiersch L (2014) Usefulness of brachial artery flow-mediated dilation to predict long-term cardiovascular events in subjects without heart disease. Am J Cardiol 113:162–7. 10.1016/j.amjcard.2013.08.051 24169007 10.1016/j.amjcard.2013.08.051

[45] Kyle UG, Bosaeus I, De Lorenzo AD, Deurenberg P, Elia M, Gómez JM et al (2004) Bioelectrical impedance analysis–part I: review of principles and methods. Clin Nutr 23:1226–1243. 10.1016/j.clnu.2004.06.004 15380917 10.1016/j.clnu.2004.06.004

